# Supplementary material for: MetaRibo-Seq measures translation in microbiomes
Source: Nat Commun. 2020 Jun 29;11:3268. doi: 10.1038/s41467-020-17081-z (PMC7324362; doi:10.1038/s41467-020-17081-z)
Supplement: Supplementary file 10 — Supplementary Data 7 [file 41467_2020_17081_MOESM10_ESM.zip › File2/Confidence_High_Taxonomy/60467_out.krona.html]

Javascript must be enabled to view this page.

members
magnitude
magnitudeUnassigned
count
unassigned
taxon
rank

60467\_out

10

2
10
superkingdom

10
phylum
1239

186801
10
class

10
order
186802

186803
5
family


SRS014613\_contig\_number\_contig-100\_118.71199SRS047014\_contig\_number\_7908
1898203
2
species

genus
3
1843210

1527
3
species

SRS014979\_contig\_number\_23301SRS024663\_contig\_number\_11054SRS065504\_contig\_number\_27265


SRS014923\_contig\_number\_39002SRS016095\_contig\_number\_18009SRS075078\_contig\_number\_contig-100\_18824.18824SRS142781\_contig\_number\_contig-100\_23354.23355SRS146812\_contig\_number\_32307
species
5
1897042
